# Impacto deletéreo del síndrome coronario agudo en la independencia del adulto mayor

**DOI:** 10.47487/apcyccv.v6i1.454

**Published:** 2025-02-12

**Authors:** Paul Coello, Inti Chaves, Paul Pacheco, Fabricio Alverca, Cristian M. Garmendia

**Affiliations:** 1 Hospital Privado Modelo, Buenos Aires, Argentina. Hospital Privado Modelo Buenos Aires Argentina; 2 Hospital Italiano de Buenos Aires, Buenos Aires, Argentina. Hospital Italiano de Buenos Aires Buenos Aires Argentina

**Keywords:** Síndrome Coronario Agudo, Dependencia, Anciano, Acute Coronary Syndrome, Functional Status, Elderly

## Abstract

**Objetivo.:**

Evaluar el rol pronóstico de la dependencia funcional en adultos mayores hospitalizados por síndrome coronario agudo (SCA) y las implicancias del evento coronario en la independencia durante el seguimiento.

**Materiales y métodos.:**

Estudio unicéntrico observacional de cohorte prospectivo en pacientes ≥65 años hospitalizados por SCA con (SCACEST) o sin (SCASEST) elevación del segmento ST en 2022. Se excluyeron aquellos con dependencia total o sin red de cuidados. La dependencia multidimensional se evaluó mediante las escalas de Barthel y Lawton y Brody el día del ingreso al centro médico, a los 30 días y al año. Se analizó como objetivo primario la asociación entre la dependencia inicial y los eventos adversos cardiovasculares mayores (MACE), así como el impacto del SCA en la dependencia a corto y largo plazo.

**Resultados.:**

Se incluyeron 110 pacientes mayores de 65 años (edad promedio 78,8±4,6 años; 61,8% hombres). Al ingreso, el 94,3% presentó dependencia funcional leve según Barthel y un grado similar en Lawton y Brody. A los 30 días, el deterioro funcional fue significativo (Barthel: 71,2±11,3; p<0,001; Lawton: 4,8±2,5; p=0,02), persistiendo al año. La dependencia inicial no se asoció con MACE. El SCACEST fue un predictor independiente del detrimento de la independencia funcional a corto plazo (OR ajustado 1,75; p=0,04).

**Conclusiones.:**

En adultos mayores con SCA, la dependencia inicial no predijo eventos adversos, pero el deterioro en la independencia funcional fue relevante, especialmente tras SCACEST. Esto destaca la importancia de estrategias personalizadas en esta población vulnerable.

## Abstract (en)

**Objectives.:**

To evaluate the prognostic role of functional status in older adults hospitalized for acute coronary syndrome (ACS) and the implications of the coronary event on functional decline during follow-up.

**Materials and Methods.:**

This was a single-center, prospective cohort study including patients aged ≥65 years hospitalized for ACS with (STEMI) or without ST-segment elevation in 2022. Patients with total dependence or lacking a care network were excluded. Functional status was assessed using the Barthel Index and the Lawton and Brody scales at admission, 30 days, and one year post-discharge. The association between initial functional status and major adverse cardiovascular events (MACE) was analyzed, as well as the impact of ACS on functional status over the short and long term.

**Results.:**

A total of 110 patients older than 65 years were included (mean age 78.8±4.6 years; 61.8% male). At admission, 94.3% presented mild functional dependence according to the Barthel Index, with similar findings on the Lawton and Brody scales. At 30 days, a significant functional decline was observed (Barthel: 71.2±11.3, p<0.001; Lawton: 4.8±2.5, p=0.02), which persisted at one year. Initial functional status was not associated with MACE. ST-segment elevation ACS (STEMI) was an independent predictor of short-term functional decline (adjusted OR 1.75; p=0.04).

**Conclusions.:**

In older adults with ACS, initial functional status did not predict adverse events; however, significant functional decline was observed, particularly after STEMI. This underscores the importance of personalized strategies for managing this vulnerable population.

## Introducción

Los síndromes coronarios agudos (SCA) con (SCACEST) y sin (SCASEST) elevación del segmento ST constituyen una de las principales causas de morbimortalidad cardiovascular a nivel global, representando una situación clínica de emergencia que requiere intervención inmediata para preservar la viabilidad miocárdica y mejorar los desenlaces a corto y largo plazo ^1^. En Argentina, la carga de enfermedad atribuida a los SCA ha ido en aumento debido a la transición epidemiológica, caracterizada por el envejecimiento poblacional y el incremento de factores de riesgo cardiovasculares en edades avanzadas ^2^. En este contexto, los pacientes adultos mayores representan un grupo particularmente vulnerable debido a la coexistencia de comorbilidades, fragilidad y menor reserva funcional.

La evaluación geriátrica integral (EGI) es una herramienta multidimensional que permite analizar aspectos médicos, funcionales, psicológicos y sociales del adulto mayor, proporcionando un enfoque holístico para la toma de decisiones clínicas ^3^. Su aplicación en el ámbito cardiovascular ha demostrado ser útil para estratificar el riesgo, predecir desenlaces adversos y guiar intervenciones individualizadas ^4^. Sin embargo, la implementación de esta estrategia en pacientes que cursan un SCA sigue siendo limitada, particularmente en países de ingresos medios como Argentina, donde los recursos disponibles y las barreras estructurales pueden dificultar su uso sistemático.

Diversos estudios han demostrado que las personas mayores con fragilidad o dependencia funcional concomitante tienen mayor riesgo de ocurrencia de eventos clínicos adversos, prolongación de la estadía hospitalaria y un mayor detrimento de parámetros vinculados con la calidad de vida en el seguimiento a mediano plazo pos-SCA ^5^. A pesar de ello, los algoritmos terapéuticos en el SCA suelen estar orientados por protocolos basados principalmente en ensayos clínicos que excluyen a pacientes geriátricos o frágiles.

El objetivo del presente estudio fue analizar las implicancias clínicas de la valoración inicial de la dependencia funcional en pacientes adultos mayores que cursan hospitalización por un SCA, valorando su rol pronóstico en términos de la evolución clínica en el seguimiento, y los factores predictores independientes del deterioro en la independencia funcional pos-SCA.

## Materiales y métodos

### Diseño de estudio

Se realizó un estudio unicéntrico observacional de cohorte prospectivo que incluyó pacientes mayores de edad con diagnóstico de un SCACEST o SCASEST de acuerdo con los criterios preespecificados en la cuarta definición universal de infarto^6^, identificando al subgrupo con edad mayor a 65 años a fin de realizar un análisis comparativo entre los pacientes más añosos y jóvenes en términos de dependencia funcional y evolución clínica en el seguimiento. Se analizó como objetivo primario la asociación entre la dependencia inicial y los eventos adversos cardiovasculares mayores (MACE) en el seguimiento, así como el impacto del SCA en la independencia a corto y largo plazo. Como objetivo secundario, se propuso identificar los factores asociados al deterioro de la independencia en pacientes adultos mayores tras haber sufrido un SCA.

### Población de estudio

Se incluyó en el análisis a pacientes adultos con diagnóstico de SCACEST y SCASEST, identificando al subgrupo con una edad ≥ 65 años con el objetivo de representar a la población de adultos mayores con edad avanzada, incluidos durante el periodo comprendido entre enero a diciembre de 2022 pertenecientes a un centro médico polivalente de alta complejidad de Argentina. Del total de la población muestral incluida, se excluyeron aquellos pacientes cuya valoración de dependencia funcional demostró un grado de dependencia total, como así también aquellos que no contaban con una red social continente para los cuidados posteriores a la hospitalización índice.

### Variables

Se analizaron como variables de interés a las características demográficas de la población en estudio, como el sexo, la edad, el índice de masa corporal (IMC), el grado de dependencia funcional y también la carga de comorbilidades cardiovasculares concomitantes, como la presencia de hipertensión arterial, dislipemia, tabaquismo activo o pasado, diabetes *mellitus* y su tratamiento farmacológico, enfermedad renal crónica (ERC), fracción de eyección ventricular izquierda y el tipo de SCA (SCACEST vs. SCASEST). En términos de las características anatómicas y del procedimiento de revascularización índice, se analizó el grado de severidad de la enfermedad coronaria ateroesclerótica de acuerdo con la puntuación de SYNTAX ^7^, la presencia o ausencia de revascularización completa, arteria coronaria culpable del evento coronario índice, el tiempo total de isquemia y el tiempo total de estadía hospitalaria posterior al procedimiento de revascularización implementado.

### Definición de términos

- Puntuación de SYNTAX (SS): medida cuantitativa reproducible de la complejidad y severidad de la enfermedad coronaria, según: SS=0-21, «baja complejidad anatómica»; SS=22-32, «complejidad anatómica intermedia», SS≥33, «elevada complejidad anatómica» ^7^.
- Grado de flujo coronario: se determinó mediante la clasificación de flujo coronario TIMI (Thrombolysis in Myocardial Infarction), según: TIMI=0, «oclusión total sin perfusión anterógrada»; TIMI=1, «penetración más allá del sitio de obstrucción coronaria sin perfusión del lecho vascular distal»; TIMI=2, «perfusión parcial distal a la obstrucción, con flujo anterógrado y aclaramiento lento del material de contraste»; TIMI=3, «perfusión completa, con flujo anterógrado y aclaramiento rápido del material de contraste» ^8^.
- Vaso culpable del evento coronario índice: se definió a aquel vaso coronario con evidencia angiográfica de oclusión luminal trombótica total o subtotal, y correlación topográfica por electrocardiografía de supradesnivel del segmento ST.
- Sangrado: se clasificó la severidad de los eventos hemorrágicos según la escala de BARC (Bleeding Academic Research Consortium), contemplando para el presente estudio los episodios de sangrado BARC ≥3 ^9^.
- Escala de Barthel: es una herramienta de evaluación que se utiliza para medir la capacidad funcional de un individuo focalizándose más en la esfera de desempeño físico, específicamente en relación con su independencia en actividades de la vida diaria (AVD). Este índice es especialmente útil en el contexto de la rehabilitación geriátrica y en pacientes con discapacidades físicas.

El índice evalúa diez actividades, que son: alimentación, baño, vestido, control de esfínteres, transferencias (de la cama a una silla y viceversa), movilidad en la cama, uso del inodoro, subir y bajar escaleras, caminar y desplazamiento en silla de ruedas, respectivamente. Cada actividad recibe una puntuación que va de 0 (dependencia total) a 10 o 15 (dependencia mínima o independencia total), dependiendo de la actividad. Al sumar los puntos, se obtiene un total que indica el nivel de autonomía de la persona. Un puntaje más alto indica un mayor nivel de independencia, siendo un puntaje de <20 subrogante de dependencia total, y un puntaje de 100 la independencia total ^10^.

- Escala de Lawton: también conocida como la «Escala de Lawton y Brody», es una herramienta de evaluación utilizada para medir la capacidad funcional de las personas mayores en relación con las AVD instrumentales.

A diferencia del índice de Barthel, que evalúa las actividades básicas, la escala de Lawton se centra en tareas más complejas que son necesarias para vivir de manera independiente en la comunidad.

La escala evalúa ocho áreas: uso del teléfono, ir de compras, preparación de alimentos, tareas del hogar, lavado de ropa, transporte, administración de medicamentos y manejo de finanzas. Cada actividad se califica con un puntaje de 0 (dependencia total) a 1 (independencia total). Un puntaje total superior indica una mayor capacidad para realizar actividades instrumentales de la vida diaria, siendo que la máxima dependencia estaría marcada por la obtención de cero puntos, y 8 puntos expresarían una independencia total ^11^.

### Procedimientos o intervenciones

Todos los pacientes fueron tratados de acuerdo a las recomendaciones de las Guías de Manejo Clínico tanto nacionales como internacionales para el manejo del SCACEST y SCASEST, que incluyó el tratamiento médico farmacológico conservador con administración de agentes fibrinolíticos, la angioplastia transluminal coronaria (ATC) percutánea y la cirugía de revascularización miocárdica (CRM), según la disponibilidad de recursos en cada centro y a la decisión del equipo médico tratante para cada caso clínico individual ^12^^-^^15^. Se evaluó la dependencia funcional el día de ingreso al centro médico por SCA, empleando escalas validadas para este fin, como la escala de Barthel y la escala de Lawton y Brody. La caracterización fue realizada por un equipo médico especializado en medicina interna y geriatría. Posteriormente, se repitió esta evaluación a los 30 días y a un año del egreso hospitalario a fin de valorar el detrimento en la independencia funcional vinculada al SCA. Esta valoración longitudinal se realizó mediante consultas presenciales en el área de control ambulatorio del centro médico, siendo llevada a cabo por el mismo equipo de médicos que realizó la evaluación inicial durante la hospitalización.

### Aspectos éticos

El estudio fue aprobado por los comités de ética del centro participantes, y se cumplió con los principios éticos establecidos en la Declaración de Helsinki, las Buenas Prácticas Clínicas y la normativa vigente en materia de ética de la investigación. Los pacientes dieron su consentimiento informado por escrito antes de ser incluidos en el estudio, al momento del ingreso sanatorial y luego del diagnóstico del SCA, el cual fue obtenido a partir del equipo médico involucrado en el presente estudio. Se explicaron los beneficios y potenciales riesgos vinculados con los procedimientos diagnósticos y terapéuticos por realizarse durante la hospitalización. La inclusión en el presente estudio no modifico la conducta terapéutica implementada. Se garantizó la confidencialidad de los datos personales y clínicos de los participantes, así como el uso exclusivo de la información para los fines de investigación establecidos.

### Análisis de datos

Las variables continuas se expresaron como media y desvío estándar o como mediana y rango intercuartílico, según las características de su distribución. La normalidad de las variables fue evaluada mediante las pruebas de Kolmogorov-Smirnov o Shapiro-Wilk, según correspondiera. Las variables categóricas se presentaron como frecuencias absolutas y porcentajes, y se analizaron mediante la prueba de chi cuadrado o la prueba exacta de Fisher, según la pertinencia.

Las comparaciones de variables numéricas se realizaron utilizando la prueba t de Student o la prueba U de Mann-Whitney, según la distribución de estas. Para evaluar la asociación entre el estado de dependencia funcional multidimensional de los pacientes al momento del ingreso sanatorial, medido mediante las escalas preespecificadas, y la ocurrencia de eventos clínicos adversos durante el seguimiento, se utilizó un modelo multivariado de regresión logística. Dicho modelo ajustó la asociación por posibles factores de confusión predefinidos (edad, sexo, IMC, diabetes *mellitus*, SCACEST, SCASEST, ERC), expresando los resultados como odds ratio (OR) con un intervalo de confianza 95% (IC 95%). Se analizaron como eventos clínicos adversos al combinado de MACE, compuesto de muerte por causa cardiovascular (CV), nuevo infarto agudo de miocardio (IAM), accidente cerebrovascular (ACV) y requerimiento de nueva revascularización secundaria a isquemia a 30 días y a un año desde el momento del egreso sanatorial.

Con el objetivo de analizar la evolución de la puntuación de las escalas de Barthel y Lawton y Brody entre el día del ingreso sanatorial, los 30 días y el primer año de seguimiento desde el momento de egreso sanatorial, se utilizó un modelo de medidas repetidas con análisis de varianza (ANOVA) de un factor, si las diferencias cumplían con los supuestos de normalidad y homogeneidad de varianzas. En caso contrario, se utilizó la prueba no paramétrica de Friedman. Se realizaron comparaciones *post hoc*, ajustando el nivel de significancia mediante el método de Bonferroni. Además, se evaluó el impacto del SCA en la puntuación de las escalas de Barthel y Lawton y Brody mediante modelos de regresión lineal multivariada, ajustando por potenciales variables confundidoras preespecificadas.

Se consideró estadísticamente significativo un error tipo I menor o igual al 5% (p < 0,05 a dos colas). Todos los análisis fueron realizados utilizando el *software* estadístico StataBE (StataCorp LLC, versión 18.0, College Station, Texas, EE. UU.).

## Resultados

Durante el período de estudio, se identificó 170 pacientes internados por un SCA. La edad promedio de la población muestral fue de 73,9±7,52 años, con un 60,8% de sexo masculino. Del total de la cohorte, el subgrupo de pacientes de ≥65 años representó el 64,7% (n=110).

En términos de características basales, el subgrupo de pacientes mayores presentó una mayor carga de comorbilidades cardiovasculares concomitantes, como dislipemia, hipertensión arterial y diabetes *mellitus*, en comparación con el subgrupo de pacientes más jóvenes (Tabla 1).

**Tabla 1:** Características basales de la población en estudio

Variable	Total (n=170)	Pacientes < 65 años (n=60)	Pacientes ≥ 65 años (n=110)	p ^*^
Edad (años)	73,91 ± 7,52	57,8 ± 6,23	78,84 ± 4,58	0,01
Sexo masculino	103 (60,8)	35 (58,7)	68 (61,82)	0,07
IMC	22,83 ± 5,34	25,71 ± 4,28	22,31 ± 2,56	0,89
HTA	106 (62,2)	24 (40,1)	82 (74,5)	0,01
DLP	77 (45,3)	23 (37,7)	54 (49,09)	0,02
DBT	30 (17,8)	8 (12,87)	22 (20,0)	0,04
Tabaquismo	82 (48,5)	35 (58,1)	47 (42,7)	0,02
ERC	10 (6,16)	1 (1,3)	9 (8,2)	0,04
EVP	5 (3,08)	1 (1,6)	4 (3,6)	0,05
FA	11 (6,72)	3 (4,2)	8 (7,3)	0,08
Cáncer	3 (1,73)	1 (1,6)	2 (1,8)	0,97
EPOC	46 (27,2)	13 (21,6)	33 (30,0)	0,08
MCP	4 (2,1)	1 (1,6)	3 (2,7)	0,62
FEVI	55,3 ± 5,83	52,47 ± 4,39	51,63 ± 3,61	0,94

Datos expresados en n (%), media ± DE.

* Valor de p para la comparación entre los subgrupos de pacientes mayores y menores de 65 años.

IMC: índice de masa corporal, HTA: hipertensión arterial, DLP: dislipemia, DBT: diabetes mellitus, ERC: enfermedad renal crónica, EVP: enfermedad vascular periférica, FA: fibrilación auricular, EPOC: enfermedad pulmonar obstructiva crónica, MCP: marcapasos definitivo, FEVI: fracción de eyección ventricular izquierda.

El SCASEST fue la forma clínica de presentación más frecuente, tanto en el subgrupo de pacientes mayores (76,6%), como en el de pacientes más jóvenes (67,4%). La arteria descendente anterior fue el vaso culpable más frecuente en el subgrupo de pacientes mayores (76,3%), mientras que la arteria coronaria derecha predominó en el subgrupo de pacientes jóvenes. No se observaron diferencias estadísticamente significativas en el tiempo total de isquemia entre ambos subgrupos, aunque sí se evidenció una mayor complejidad anatómica, según la puntuación del *score* de SYNTAX, en los pacientes mayores. La estrategia de revascularización mayormente utilizada en ambos subgrupos fue la ATC, seguida de la cirugía de CRM. Sin embargo, se observó un mayor tiempo de estadía hospitalaria en el subgrupo de pacientes mayores (Tabla 2).

**Tabla 2:** Presentación clínica, características anatómicas y procedimiento de revascularización realizado

Variable	Total (n=170)	Pacientes < 65 años (n=60)	Pacientes ≥ 65 años (n=110)	p ^*^
Síndrome coronario				
SCACEST	46 (27,1)	20 (33,3)	26 (23,6)	0,07
SCASEST	124 (72,9)	40 (67,4)	84 (76,6)	0,10
Tiempo de isquemia (min)	134,2 ± 32,0	134,5 ± 26,8	140,5 ± 38,1	0,14
Acceso vascular				
Radial	155 (91,1)	57 (94,3)	97 (88,3)	0,22
Femoral	15 (8,8)	3 (5,7)	13 (11,7)	0,04
Vaso culpable				
LAD	88 (51,8)	23 (38,7)	84 (76,3)	0,03
RCA	68 (39,9)	33 (54,2)	9 (8,2)	0,01
CX	14 (8,3)	4 (7,1)	17 (15,5)	0,04
SYNTAX score	22,4 ± 6,86	19,3 ± 5,7	28,7 ± 11,1	0,02
Estrategia terapéutica				
ATC	168 (98,9)	58 (97,6)	110 (100,0)	0,43
CRM	2 (1,1)	2 (3,3)	0 (0)	0,07
Flujo TIMI post ATC				
0	39 (17,1)	8 (25,0)	28 (23,9)	0,31
I	0	0 (0)	0 (0)	-
II	9 (3,9)	2 (6,3)	4 (3,4)	0,08
III	180 (78,9)	22 (68,8)	85 (72,6)	0,16
No. stents	1,33 ± 1,02	1,86 ± 1,59	1,42 ± 1,04	0,32
Longitud stents (mm)	32,15 ± 26,14	33,48 ± 26,49	31,05 ± 22,30	0,25
Estadía hospitalaria (días)	3 (1-4)	3 (1-4)	5 (2-6)	0,04

Datos expresados en n (%), media ± DE, mediana (RIQ)

* Valor de p para la comparación entre los subgrupos de pacientes mayores y menores de 65 años.

DE = desvío estándar; RIQ: rango intercuartílico, SCACEST: síndrome coronario agudo con elevación del segmento ST, SCASEST: síndrome coronario agudo sin elevación del segmento ST, LAD: arteria descendente anterior, RCA: arteria coronaria derecha, CX: arteria circunfleja, ATC: angioplastia transluminal coronaria, CRM: cirugía de revascularización miocárdica.

**Figura 1:**
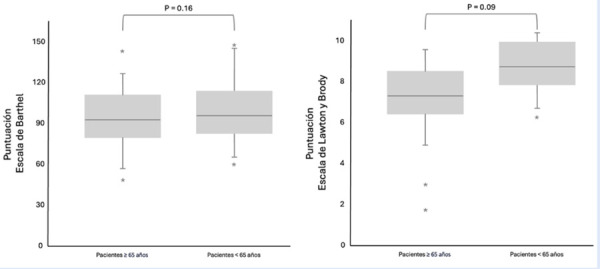
Valoración funcional inicial según las escalas de Barthel y Lawton y Brody, de acuerdo con el subgrupo etario.

En términos de valoración de la dependencia funcional al ingreso sanatorial, el subgrupo de pacientes mayores obtuvo un promedio de puntuación en la escala de Barthel de 94,63±17,42 puntos, clasificándose predominantemente con un grado de dependencia funcional leve. Así, del total de pacientes mayores, el 94,3% (n=103) presentó dependencia leve, el 5,6% (n=6) dependencia moderada, y el 0,1% (n=1) dependencia funcional severa, respectivamente. No se observaron diferencias estadísticamente significativas en relación con la puntuación de la escala de Barthel entre el subgrupo de pacientes mayores y el de pacientes más jóvenes al momento del ingreso sanatorial (Figura 1).

En relación con la valoración de la dependencia funcional mediante la escala de Lawton y Brody, el subgrupo de pacientes mayores obtuvo un promedio de 7,22±1,36 puntos, identificándose también con un grado de dependencia leve. En comparación con los pacientes más jóvenes, se observó una tendencia no estadísticamente significativa hacia una menor dependencia funcional en el subgrupo de pacientes mayores (Figura 1).

En términos del rol pronóstico de la valoración de la dependencia funcional al momento del ingreso hospitalario en términos de la evolución clínica en el seguimiento a 30 días, y analizando esta asociación en relación con potenciales factores confundidores, no se observó una asociación estadísticamente significativa entre la puntuación de la escala de Barthel con una mayor ocurrencia del combinado de eventos clínicos adversos en el seguimiento a 30 días desde el evento coronario índice (MACE, OR ajustado 1,03 [IC 95% 0,10-2,38]; p=0,89). Un hallazgo similar se observó en relación con la valoración funcional mediante la escala de Lawton y Brody, no identificándose una asociación estadísticamente significativa entre la valoración funcional al ingreso sanatorial con la evolución clínica en el seguimiento a 30 días (MACE, OR ajustado 1,06 [IC 95% 0,09-3,52]; p=0,96). A su vez, resultados comparables se evidenciaron en la evolución clínica alejada a un año de seguimiento, sin observarse un incremento estadísticamente significativo del combinado clínico vinculado con la valoración de la dependencia funcional inicial, tanto por la escala de Barthel (MACE, OR ajustado 1,28 [IC 95% 0,54-5,19]; p=0,68), como por la escala de Lawton y Brody (MACE, OR ajustado 1,12 [IC 95% 0,19-4,32]; p=0,71), respectivamente.

**Figura 2:**
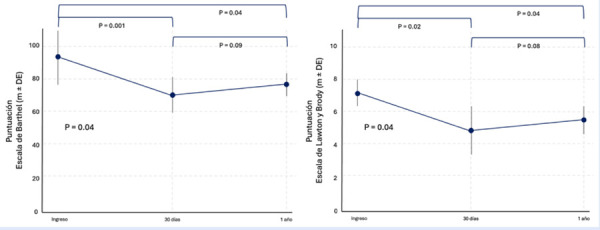
Valoración funcional longitudinal según la escala de Barthel y Lawton y Brody luego de un SCA.

Con un seguimiento a 30 días desde el evento coronario índice, la valoración de la dependencia funcional de acuerdo con la puntuación de la escala de Barthel en el subgrupo de pacientes mayores obtuvo un valor de 71,24±11,36 puntos, caracterizando a este subgrupo con un grado de dependencia leve, y evidenciando un detrimento estadísticamente significativo en relación con la valoración funcional al momento del ingreso sanatorial (94,63±17,42 vs. 71,24±11,36; p<0,01). A su vez, esta valoración realizada a un año de seguimiento desde el SCA obtuvo una puntuación de 77,85±7,49 puntos, identificándose una reducción estadísticamente significativa en relación con la valoración al momento de la hospitalización índice (94,63±17,42vs. 77,85±7,49; p<0,01), y una tendencia ascendente no significativa en relación con la valoración funcional realizada a 30 días de seguimiento (71,24±11,36 vs. 77,85±7,49; ANOVA de medidas repetidas p=0,04). Hallazgos similares se observaron al realizar la valoración funcional mediante la escala de Lawton y Brody, observándose una reducción estadísticamente significativa de la independencia funcional a 30 días de seguimiento desde el evento coronario (7,22±1,36 vs. 4,83±2,54; p=0,02), y a un año de seguimiento (7,22±1,36 vs. 5,64±1,68), identificándose una tendencia ascendente no estadísticamente significativa entre el periodo de 30 días a un año, respectivamente (4,83±2,54 vs. 5,64±1,68; ANOVA de medidas repetidas p=0,04) (Figura 2).

**Figura 3:**
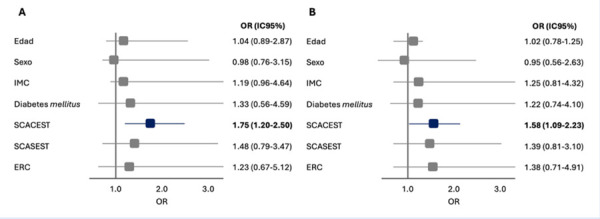
Implicancias clínicas del SCA en la valoración funcional del adulto mayor a 30 días desde el evento coronario índice. A. Valoración por escala de Barthel, B. Valoración por escala de Lawton y Brody.

Mediante un análisis multivariado por regresión logística y ajustado por factores potencialmente confundidores se identificó al SCACEST como un parámetro significativamente asociado con el deterioro de la independencia funcional a los 30 días de seguimiento. Esta asociación fue evidente tanto al evaluar la funcionalidad mediante la escala de Barthel (OR ajustado 1,75 [IC 95% 1,20-2,50]; p=0,04), como mediante la escala de Lawton y Brody (OR ajustado = 1,58 [IC 95% 1,09-2,23]; p=0,04) (Figura 3).

## Discusión

De acuerdo con los resultados obtenidos en el presente estudio, se destacan los siguientes hallazgos: en pacientes añosos con un SCA, la valoración de la independencia funcional multidimensional realizada al momento del ingreso sanatorial, utilizando escalas validadas y específicamente diseñadas para tal fin, no demostró un papel predictor en relación con la ocurrencia de eventos clínicos adversos durante el seguimiento.

La falta de un rol pronóstico claro de la valoración de la independencia funcional inicial puede atribuirse a varios factores. Las escalas de Barthel y Lawton y Brody se enfocan principalmente en evaluar la capacidad del paciente para realizar actividades cotidianas, como movilidad, higiene, alimentación y autocuidado. Inicialmente, la escala de Barthel fue desarrollada para evaluar la gravedad de la discapacidad en pacientes con trastornos neuromusculares y musculoesqueléticos, cuya enfermedad afectaba el movimiento independiente de las extremidades. No obstante, pronto se convirtió en una herramienta ampliamente utilizada para valorar los cambios funcionales en la rehabilitación de personas que habían sufrido un ACV ^16^. Dado que el ACV y los SCA comparten ciertos mecanismos fisiopatológicos, resulta razonable suponer que estas escalas podrían desempeñar un papel pronóstico en este contexto clínico. A su vez, la escala de Lawton y Brody ha demostrado ser útil como herramienta predictora de riesgo de eventos clínicos adversos en múltiples escenarios clínicos que involucran población de adulto mayor, como pacientes portadores de deterioro neurocognitivo ^17^ o cáncer oncohematológico ^18^, lo cual la posiciona como herramienta útil para la predicción de riesgo en esta subpoblación de adulto mayor con SCA por el detrimento físico que este conlleva. Asimismo, podrían constituir una herramienta válida para determinar el impacto deletéreo en términos de dependencia funcional en adultos mayores con SCA, tanto en el seguimiento a mediano como a largo plazo.

Aunque son útiles para medir la independencia funcional, pueden no reflejar la complejidad de las condiciones clínicas subyacentes de los pacientes con SCA, como comorbilidades, deterioro cognitivo, fragilidad y características fisiopatológicas, que pueden influir más significativamente en la aparición de eventos clínicos adversos. Sin embargo, tales condiciones no siempre son suficientemente captadas por las escalas de valoración funcional, que se enfocan principalmente en la capacidad de realizar tareas cotidianas sin tener en cuenta aspectos complejos de la salud física y mental. Este contexto pone de manifiesto la necesidad de una evaluación más integral del estado clínico de los pacientes, que abarque no solo la dependencia funcional, sino también factores como el estado cognitivo, la fragilidad y las condiciones comórbidas. 

En este escenario, existe robusta evidencia que la presencia de deterioro cognitivo y fragilidad concomitante se asocia a una peor evolución clínica en pacientes añosos cursando hospitalización por un SCA ^19^^-^^20^; sin embargo, y a pesar de haberse demostrado un rol pronóstico de la valoración funcional de los pacientes añosos en otros escenarios clínicos ^21^^,^^22^, hasta la fecha existe escasa evidencia científica que haya valorado el rol pronóstico y las implicancias clínicas del estado funcional en pacientes añosos cursando hospitalización por un SCA ^23^. Así, y aunque la valoración funcional es una herramienta útil para comprender el grado de dependencia de los pacientes y orientar el manejo rehabilitador, su capacidad predictiva de eventos clínicos adversos durante el seguimiento en pacientes mayores con SCA parece ser limitada. Asimismo, se subraya la importancia de considerar una evaluación más holística que combine la valoración funcional con otras mediciones, como el estado cognitivo, la fragilidad y las características específicas del SCA a fin de mejorar la predicción de los resultados clínicos en esta población vulnerable.

Este estudio identifico al SCACEST como un factor estrechamente vinculado con una reducción en la independencia funcional a los 30 días tras el evento coronario índice, hallazgo que se mantuvo a lo largo del año de seguimiento. No obstante, no se observaron cambios significativos entre los 30 días y el año, lo que sugiere que el impacto clínico del SCACEST en la independencia funcional es más pronunciado en el corto plazo. En los pacientes mayores, la reserva funcional cardiovascular es más limitada debido a los efectos acumulativos del envejecimiento y las comorbilidades cardiovasculares concomitantes, lo que aumenta el riesgo de una recuperación más lenta y de un empeoramiento de la independencia funcional ^24^^,^^25^. Además, el SCA puede contribuir a un proceso de depleción física generalizada, ya que la hospitalización prolongada, las intervenciones médicas diagnósticas y terapéuticas realizadas durante la hospitalización índice o las complicaciones intrahospitalarias periprocedimiento pueden llevar a una inmovilización relativa o al desarrollo de debilidad muscular. En este contexto, el desacondicionamiento físico asociado con la inmovilidad y el reposo prolongado contribuye al deterioro funcional, especialmente en los pacientes ancianos, quienes ya presentan una disminución en la masa muscular y la fuerza debido a los efectos naturales del envejecimiento ^26^^,^^27^. 

Por otro lado, el impacto psicosocial también cumple una función importante. La hospitalización por un SCA en pacientes mayores no solo implica un desafío físico, sino también emocional. La ansiedad, el estrés y la depresión pueden desencadenarse como resultado de la enfermedad aguda, lo cual puede interferir en la motivación del paciente para participar en actividades de rehabilitación o en el cumplimiento de las recomendaciones médicas ^28^. El deterioro cognitivo y la fragilidad, características comunes en este grupo etario, también se pueden ver exacerbadas por el estrés de un evento cardiovascular, lo que agrava el impacto en la capacidad funcional. Finalmente, la medicación y el tratamiento pos-SCA, como el uso de anticoagulantes, betabloqueantes o inhibidores de la enzima convertidora de angiotensina, también pueden tener efectos secundarios que afecten la capacidad física del paciente, como la hipotensión ortostática ^29^, mareos o fatiga ^30^, contribuyendo al deterioro funcional durante los primeros meses tras el evento.

Este estudio presenta limitaciones inherentes a su diseño observacional, lo que impide establecer relaciones causales directas entre la valoración de la independencia funcional y los eventos clínicos adversos durante el seguimiento. Factores no medidos o no controlados podrían haber influido en los resultados. La inclusión de pacientes con comorbilidades y fragilidad introdujo heterogeneidad, lo que limita la generalización de los hallazgos a otras poblaciones. Además, las escalas de valoración funcional, aunque validadas, se enfocan en aspectos específicos de la vida diaria y no consideran dimensiones como el estado cognitivo o la fragilidad, que podrían tener un mayor impacto en la predicción de eventos adversos. La falta de un seguimiento más detallado de variables adicionales, como la rehabilitación post-evento y los efectos psicosociales, también limita la comprensión completa de los factores que influyen en la capacidad funcional y en la ocurrencia de eventos adversos.

En conclusión, en pacientes añosos hospitalizados por un SCA, la valoración geriátrica de la independencia funcional al ingreso hospitalario no predice eventos clínicos adversos durante el seguimiento. Sin embargo, el SCACEST está asociado con una reducción de la independencia multidimensional a los 30 días, y este deterioro persiste durante el año siguiente. Estos hallazgos resaltan la importancia de abordar tanto los aspectos cardiovasculares como los factores que afectan la independencia en esta población vulnerable, promoviendo intervenciones tempranas y estrategias de manejo integral.

## Fuente de financiamiento:

Autofinanciado
